# Association of global and regional central circulation transit time with left ventricular end diastolic pressure using dynamic magnetic resonance imaging

**DOI:** 10.1186/1532-429X-14-S1-P288

**Published:** 2012-02-01

**Authors:** Laura Y Li, Jeannette McLaughlin, Peter D Rhee, Jie J Cao

**Affiliations:** 1 Research, St. Francis Hospital, Roslyn, NY, USA

## Background

Prolonged central circulation transit time (TT) has long been associated with heart failure and left ventricular (LV) dysfunction. However, it is not known which portion of the central circulation is associated with this prolongation. Moreover, the relation of global and regional TT to LV end diastolic pressure (LVEDP) is not defined. In this study we assessed the global and regional TT in each portion of the central circulation and investigated their relations to LVEDP in patients undergoing cardiac catheterization.

## Methods

All patients were prospectively recruited and underwent cardiac MRI within 5 hours of cardiac catheterization. Dynamic MR images were acquired during the infusion of gadopentetate dimeglumine (0.01 mmol/kg) at 6 ml/s. Areas of interest were drawn and propagated through phases in the right atrium (RA), right ventricle (RV), main pulmonary artery (PA), left and right lung, left atrium (LA), LV and ascending aorta to construct time-intensity curves. TT was defined as the time between the peaks of time-intensity curves. All TTs were normalized to RR intervals. Central circulation TT was defined as TT from RA to aorta, while regional TT was defined as RA to RV, RV to PA, PA to lungs (averaged value of the left and right lung), lungs to LA, LA to LV, and LV to aorta. LVEDP was assessed during cardiac catheterization.

## Results

Of the 48 subjects studied the mean age was 59 years and 32 (67%) were male. Average LV ejection fraction (LVEF) was 48±15% and the LVEDP 15±8 mmHg. Central TT was significantly prolonged in patients with elevated LVEDP (>12 mmHg) 15.7±5.8 cardiac cycles vs. 10.5±2.2 cardiac cycles (p=0.002) in those with normal LVEDP (≤12 mmHg). Regional analysis demonstrated that TT prolongation was present in PA to lungs, lungs to LA, LA to LV and LV to aorta but not in RA to RV and RV to PA. The multivariate regression model which included all regional TTs as covariables showed that TT from lung to LA had the strongest association with LVEDP (beta coefficients 0,424, p<0.001) followed by TT from LA to LV (0,325, p=0.007) and TT from PA to lungs (0,240, p=0.037). TTs from other regions were not significantly associated with LVEDP.

## Conclusions

Central and regional circulation TT can be assessed by dynamic MR imaging. Central TT was significantly prolonged in patients with elevated LVEDP largely due to regional TT prolongation in lungs to LA, LA to LV and PA to lungs circulation.

## Funding

None.

